# Vacuole dynamics and popping-based motility in liquid droplets of DNA

**DOI:** 10.1038/s41467-023-39175-0

**Published:** 2023-06-16

**Authors:** Omar A. Saleh, Sam Wilken, Todd M. Squires, Tim Liedl

**Affiliations:** 1 Materials Department and Physics Department, University of California, Santa Barbara, CA 93106 USA; 2 Chemical Engineering Department, University of California, Santa Barbara, CA 93106 USA; 3 Physics Department, Ludwig-Maximilians University, Munich, Germany

**Keywords:** Self-assembly, Bioinspired materials

## Abstract

Liquid droplets of biomolecules play key roles in organizing cellular behavior, and are also technologically relevant, yet physical studies of dynamic processes of such droplets have generally been lacking. Here, we investigate and quantify the dynamics of formation of dilute internal inclusions, i.e., vacuoles, within a model system consisting of liquid droplets of DNA ‘nanostar’ particles. When acted upon by DNA-cleaving restriction enzymes, these DNA droplets exhibit cycles of appearance, growth, and bursting of internal vacuoles. Analysis of vacuole growth shows their radius increases linearly in time. Further, vacuoles pop upon reaching the droplet interface, leading to droplet motion driven by the osmotic pressure of restriction fragments captured in the vacuole. We develop a model that accounts for the linear nature of vacuole growth, and the pressures associated with motility, by describing the dynamics of diffusing restriction fragments. The results illustrate the complex non-equilibrium dynamics possible in biomolecular condensates.

## Abstract (web summary)

In this work, the authors report the appearance of vacuoles within the droplets of an enzyme-driven biomolecular liquid. The mechanisms of these behaviors are quantitatively explained with a diffusive capture model of enzyme dynamics.

## Introduction

The phase separation of biomolecules into spatially-separated dilute and condensed phases has been shown to control the formation of a broad range of cellular organelles^1–5^. Such processes also have technological implications, as they permit the formation of micron scale particles with applications in cosmetics or drug delivery^6^. It is thus of interest to study and quantify the behavior of micron-scale phase-separated condensates, and particularly their behavior in the complex multi-component situations that are physiologically relevant.

Sequence-engineered DNA self-assembly offers an attractive platform for creating model systems of condensate behavior, as it allows nanoscale control of biomolecular interactions, both among the particles that constitute the condensate, and between those particles and external solutes, notably including sequence-sensitive transactions between proteins and DNA. Indeed, building on foundational work in the creation of DNA-based materials^7–10^, prior work has shown that liquid droplets of DNA can be reliably formed through the creation and self-association of multi-armed DNA “nanostar” particles^11–13^. Nanostars consist of three or four double-stranded arms connected at a flexible junction, and terminating in a single-stranded sticky end^14,15^. The sticky ends control attractive hybridization interactions between particles that drive nanostar condensation into gel or liquid phases^11,12^. Such materials have been characterized in terms of their phase and physical properties^12,13,15–19^, and have been engineered to display various sequence-specific dynamic behaviors driven by other nucleic strands or proteins^12,13^.

Previously, we have developed a model system for precision study of solute/condensate dynamics consisting of DNA nanostar droplets that are degraded by DNA-cleaving restriction enzymes^20^. A notable finding was that the enzymes can penetrate the droplets, and drive the formation of relatively dilute internal inclusions, here termed vacuoles, that undergo multiple cycles of growth and bursting. Vacuole growth was attributed to the osmotic pressure of the DNA fragments that are created by enzymatic cleavage reactions. Vacuole or bubble formation is a general property of liquid phases, and has been observed in other biomolecular condensate systems^21–23^. Typically, though, such inclusions are formed as a result of re-equilibration following spatially uniform, bulk changes in thermodynamic parameters, such as the changes in pressure that drive bubble formation in decompression sickness (“the bends”)^24^. In contrast, vacuole formation in the enzyme/DNA droplet system is a localized, non-equilibrium, “active” phenomenon driven by catalyzed conversion of chemical energy into the work required to expand the vacuole. Such localized, dissipative phenomena are of clear biological interest, as emphasized by a recent study of a model system of mitochondrial condensates in which vacuole-like inclusions were formed through the action of a localized enzymatic process (transcription)^25^.

Here, we investigate active, enzymatic vacuole formation in a DNA droplet. Through quantification of vacuole growth trajectories, we show that vacuole dynamics reflect the balance between the vacuole’s internal osmotic pressure and the interfacial tension of the vacuole/DNA liquid. We further find that vacuole popping creates bursts of droplet motility, with speeds up to ≈ 100 nm/s. We show that the forces driving these motions are consistent with those arising from the pressures predicted by our model, and use the model to provide estimates of the dissipative energetics underlying the dynamics. Generally, this work highlights the complex mix of osmotic and mechanical effects that can occur in non-equilibrium condensate systems, as well as demonstrating a popping-based motility mechanism driven by jetting at low Reynolds number.

## Results

### Nanostar design and droplet assembly

**Fig. 1:**
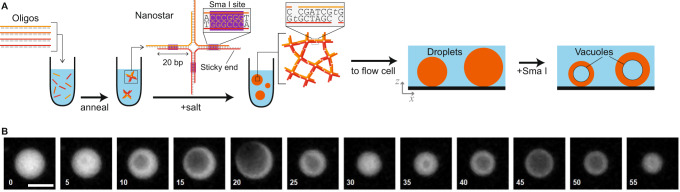
Experimental overview. **A** Multi-armed DNA nanostar particles, containing a restriction enzyme cleavage site in the arms, are assembled by annealing a solution of DNA oligomers, one of which carries a fluorophore. The nanostars are induced to form droplets by raising the solution salt concentration. Droplets are transferred to a flow cell, then cleavage is instigated by adding the enzyme, which causes both droplet shrinkage and the appearance of vacuoles, as revealed by fluorescent microscopy. Schematic adapted from ref. ^20^. **B** Representative images from a time-lapse acquisition showing a droplet of nanostars undergoing two cycles of vacuole growth and popping due to the action of the enzyme Sma I; see also Supplementary Movie 1. Each image is labeled with the time in minutes; scale bar 40 μm.

The DNA nanostars used here match those used in previous work^20^. Briefly, DNA sequences were designed using the NUPACK software^26^, and following guidelines for promoting self-assembly of a liquid phase^11,15^ (full sequences given in Supplementary Table 1). Each nanostar has three or four double-stranded DNA arms joined at a junction by flexible, unpaired bases, with distal ends terminating in a 6-base palindromic sticky end (Fig. 1). Nanostar arms contain the recognition sequence for Sma I, a restriction enzyme that recognizes the 6-base *GGGCCC* duplex sequence, and cleaves through the center of that sequence so as to create blunt-ended fragments. Three different nanostar designs were used in this work: a trimer, and two tetramers with different sticky-end sequences. A schematic of one type of tetramer is shown in Fig. 1. No significant differences were found for the vacuole dynamics within different nanostar liquids, and the remainder of this work presents results from all three types.

DNA droplets were formed by an annealing process, in which constituent DNA oligomers (including a small fraction labeled with a fluorescent dye) were mixed, held briefly at 90 °C, then cooled over 2 h to room temperature. Buffer conditions were then adjusted to those favorable for enzymatic activity, and the mixture was briefly stored to promote the growth of micron-scale droplets. The mixture was then added to a pre-formed glass flow-cell, enzymes were added, and the flow-cell was sealed and visualized using wide-field, time-lapse fluorescent microscopy. Droplets were driven by sedimentation to collect near the flow cell’s lower glass surface. For experiments quantifying vacuole growth, a previously-described protocol was used to lightly adhere droplets to the glass surface, in which the nanostar droplets were first strongly adhered to a glass surface with a hydrophobic coating, then partially de-adhered through competition with added BSA^20^. Experiments on motility focused on non-adhered droplets, in which the glass surface was passivated with BSA prior to droplet addition. Further details can be found in the “Methods” section.

### Appearance and linear growth of vacuoles

After the enzyme was introduced, all droplets began to shrink, eventually disappearing over time due to degradation by restriction enzymes localized on the droplet exterior^20^. In addition, certain larger droplets showed the appearance of vacuoles. Frequently, such droplets show a “bubbling” appearance, in which vacuoles displayed multiple cycles of growth and popping with a typical period of several tens of minutes (as in Fig. 1; see also Supplementary Movie 1); previous observations indicated up to 10 popping events can be seen from a single droplet^20^. We attribute vacuole formation to the effects of restriction enzymes located inside the droplet. Prior work showed that enzyme internalization can occur in this system, and attributed it to a nanostar-assisted transport process in which a restriction enzyme binds to the double-stranded portion of a nanostar, then is carried along as the nanostar diffuses through the DNA liquid^20^.

**Fig. 2:**
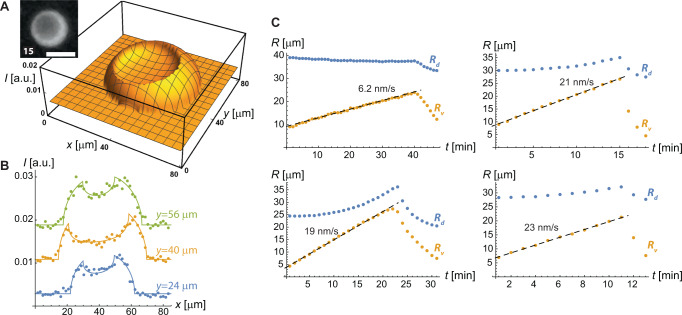
Analysis of vacuole and droplet size. **A**, **B** Example of fitting-based analysis to find vacuole and droplet sizes. Droplet images, such as shown in the inset to **A**, are fit to a “double-sphere” function (see Supplementary Discussion) accounting for the fluorescent intensity of the spherical droplet, and the lesser fluorescence of the spherical vacuole. The plot in **A** shows intensity, *I* (in arbitrary units), vs. *x* and *y* for the double-sphere function fit to the inset image (which is from the time series shown in Fig. 1; scale bar 40 μm). This particular fit indicates the droplet and vacuole radii of *R*_d_ = 27.7 ± 0.1 μm and *R*_v_ = 18.8 ± 0.1 μm. An alternate view of the same fit is shown in **B**, in which the fit (lines) is compared directly to the image data (points) for cuts along the *x* axis at three different *y* positions; the data are offset for clarity. **C** Experimental trajectories of *R*_v_ (yellow points) and *R*_d_ (blue points) for four representative vacuole growth events. The dashed line indicates the best fit to the growth phase of the vacuole, with slope (growth rate) indicated. We analyzed 44 such growth events, finding the growth rate to range between 2 and 32 nm/s, with a mean of 17 nm/s. Note the tendency of the droplet to swell (increase of *R*_d_) as the vacuole grows, indicating the presence of an internal pressure inside the vacuole. Source data are provided as a Source data file.

We analyzed vacuole growth through a fitting-based analysis of the images. In particular, the measured image intensity patterns were well fit by a “double-sphere” fitting function that described the expected intensity pattern of a sphere projected into two dimensions (corresponding to the fluorescence of the DNA liquid phase), with a second, smaller sphere subtracted from it (corresponding to the low-fluorescence interior of the vacuole). The fitting parameters consisted of the radius and position of the droplet and vacuole, along with parameters describing the fluorescent intensity of the DNA phase and of the background; the exact form of the fitting function is given in the Supplementary Discussion, and typical fitting results are shown in Fig. 2. There were generally small systematic deviations between the image data and the fit at sphere edges, which we attribute to diffraction limitations present in the imaging system that are not accounted for in the fitting function. Yet, apart from the edges, the double-sphere fitting performed well in replicating the captured intensity patterns, lending confidence to the physical interpretation of the fitting parameters.

We found the vacuole radius, *R*_v_, to consistently grow linearly in time, *R*_v_ ∝ *t*, up until the popping event (Fig. 2). Such linear growth was present for both shorter growth events that lasted 5–10 min, and for the handful of longer events that lasted up to 45 min. The vacuole growth rate, ${\dot{R}}_{{{{{{\rm{v}}}}}}}$, ranged between 2 and 32 nm/s, with an average of 17 nm/s. Vacuole popping was always followed by a relatively rapid deflation that was characterized by transient non-spherical shapes of the vacuole and/or droplet (see, e.g., the 25 minute image in Fig. 1); because of the observed asymmetries, the estimates of *R*_v_ and *R*_d_ during deflation are only approximations. The droplet radius, *R*_d_, typically first slowly decreased with time due to external enzymatic degradation, then increased when *R*_v_ approached *R*_d_, then resumed a slow decrease after the popping event (Fig. 2).

Internalized enzymes that cleave nanostars could in principle form vacuoles solely by degradation, i.e., by converting a certain volume of liquid-phase nanostars into an equal volume of solution of disconnected nanostar fragments. However, this mechanism would result in the same net concentration of DNA in the vacuole and the surrounding DNA liquid, which is inconsistent with the lower DNA concentration in the vacuole indicated by its relative darkness in the image. Further, the observed droplet swelling upon vacuole growth (increase in *R*_d_) would not occur if enzymes were solely degrading the liquid phase—instead, this demonstrates the presence of an excess internal pressure within the vacuole that drives the swelling of the droplet. We attribute this to the osmotic pressure arising from DNA restriction fragments that are at least transiently trapped in the vacuole by the liquid-phase meshwork. Such trapping is reasonable: prior work has shown that permeation of neutral solutes through the nanostar liquid becomes disallowed when they are larger than a characteristic average length scale, the mesh size, of about 8 nm^27^. The effective mesh size will be smaller for a DNA fragment that is strongly electrostatically repelled from the DNA meshwork, and likely of similar size to the ≈4 nm length of the fragments, resulting in a relatively modest rate of diffusive transport of the fragment through the liquid. Note also that the major mode of transport of restriction enzymes through the liquid meshwork (i.e., binding to and transport by nanostars^20^) is not available to the fragments.

### Popping-driven motility

**Fig. 3:**
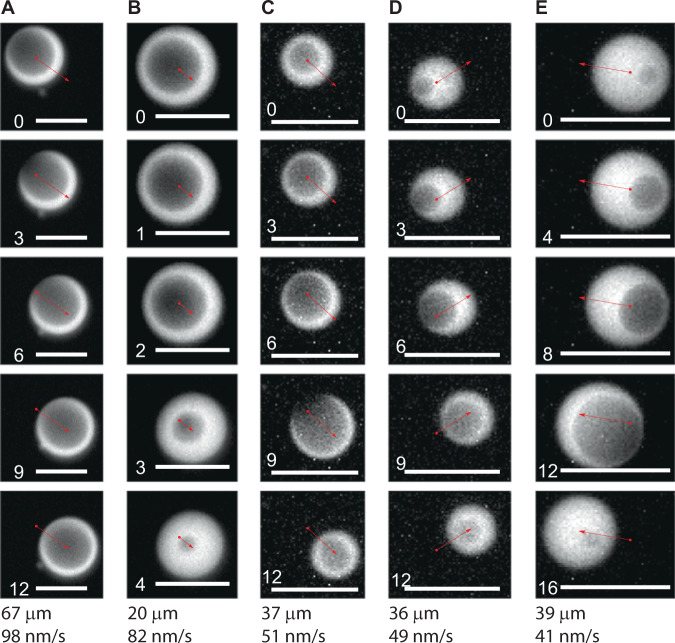
Vacuole-popping based motility. **A**–**E** Five representative time-lapse acquisitions demonstrating droplet motility powered by vacuole popping (see also Supplementary Movies 2–6). Time proceeds top to bottom in each column. Each image series shows a vacuole that is placed asymmetrically with respect to the droplet, and that bursts, moving the droplet in the opposite direction. In most cases, motion is simultaneous with a visible reduction of vacuole size. The overlaid arrows span the droplet centroid at the start and end of the burst. Each image shows an 80 μm scale bar and a time stamp in minutes; the distance traveled and average speed of the event is indicated below each column.

**Fig. 4:**
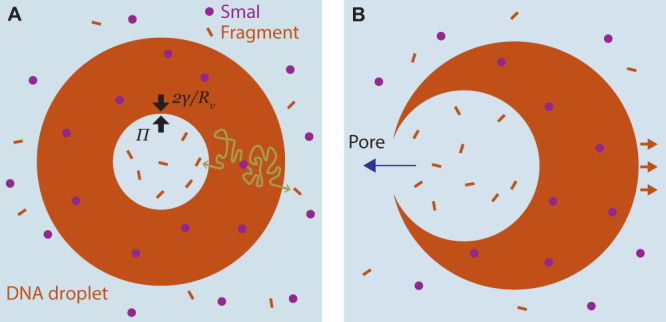
Mechanistic interpretation of vacuole growth rates and popping-based motility. **A** Schematic of the mechanism of vacuole growth consistent with the observation of linear growth, and as captured in the model (Eqs. (1)–(3)). A DNA liquid droplet, with external radius *R*_d_, is embedded with enzymes that continuously generate restriction fragments. The fragments randomly diffuse (as exemplified by the sketched tortuous paths), and exit either into the vacuole or the exterior. Fragments that accumulate in the vacuole generate an osmotic pressure, Π, that swells the vacuole against the Laplace pressure, 2*γ*/*R*_v_, where *R*_v_ is the vacuole radius, and *γ* is the interfacial tension. **B** Mechanism of vacuole popping motility. Vacuoles open a pore upon reaching the exterior droplet interface. Hydraulic pressure within the vacuole, similar in magnitude to the osmotic pressure, drives solution outflow through the pore, sending the droplet jetting in the opposite direction.

Droplets that were de-adhered from the substrate, and that exhibited vacuole formation, frequently displayed bursts of motion. In many instances, the vacuole was visibly placed towards one side of the droplet, and popping of the vacuole led to droplet motion towards the opposite side (Fig. 3 and Supplementary Movies 2–6). This implies a jetting-type propulsive mechanism in which hydraulic pressures within the vacuole drives fluid through a pore that connects the vacuole to the exterior (see Fig. 4). In this picture, the outflow through the pore would drive the droplet in the opposite direction. That said, not all vacuole popping events drove droplet motion. We attribute this to buoyancy effects: vacuoles are less dense than the surrounding DNA liquid phase, and thus will rise within the droplet, likely thinning the droplet at its highest point, i.e., opposite to the glass surface. Pore formation at that highest point would lead to jetting directly downwards into the surface, with no discernible lateral motion.

We tracked each motile droplet by calculating its intensity-weighted centroid, and analyzed the trajectories to find bursts, defined as a sequence of successive displacements that are roughly parallel (with each displacement within 60° of the previous one), and over which the droplet moved by a total of at least 20 μm (a threshold purposefully set high, to ensure removal of short Brownian motions). We identified 25 such bursts, with average velocities ranging from 29 nm/s to 176 nm/s, with a median of 51 nm/s. We then estimated the force required to produce such velocities by assuming droplet motion is impeded by Stokes drag, roughly adjusted for the presence of the nearby surface. Particularly, we set *f*_burst_ = 6*πηavα*, where *η* is the water viscosity, *a* is the average droplet radius over the course of the burst event, and *α* ≈ 3 is a drag-increase factor that accounts for the hydrodynamic effect of the glass surface, as estimated by analysis of thermal droplet diffusion (Supplementary Fig. 1). Using this, the forces ranged from *f*_burst_ = 0.02 to 0.2 pN, with a median of 0.03 pN.

The burst forces can be used to approximate the excess internal pressure within the vacuole through $p={f}_{{{{{{\rm{burst}}}}}}}/\pi {R}_{{{{{{\rm{pore}}}}}}}^{2}$, given an estimate for the size of the pore *R*_pore_. The pore size must be somewhat larger than the mesh size to permit transport of fragments, and the accompanying directed pressure release that leads to droplet motion. We do not expect the pore to be too large, though, as once a pore forms, the internal pressure will dissipate, which would presumably disfavor further pore growth and lead eventually to the observed re-sealing of the pore. Given the 8 nm mesh size, a rough estimate is then that the pore is a few hundred nanometers in size, which indicates *p* is of order 0.1 to 1 Pa.

### Modeling vacuole growth

Figure 4 depicts a hypothesized model of fragment generation and vacuole dynamics that replicates the *R*_v_ ∝ *t* observation (Fig. 2). Underlying the model is the assumption that fragments must largely be generated within the DNA liquid phase, as that is the location of high concentration of the enzymatic substrate (the DNA nanostars). Fragments created in the DNA liquid must then be transported to the vacuole, which could occur due to advection (solvent flow into the vacuole, driven by osmotic pressure) or diffusion. Fragments that accumulate in the vacuole create an osmotic pressure, Π, that is balanced by the Laplace pressure of the vacuole interface.

To determine whether advection or diffusion is the dominant mode of transport, we calculate the Peclet number, Pe = *uL*/*D*, using an advective flow rate, *u* ≈ 20 nm/s, fixed by the measured vacuole growth rate (Fig. 2); a transport length scale, *L* ≈ 10 μm, set by the typical droplet size; and a fragment diffusion constant *D* ≈ 10–100 μm^2^/s. The upper limit of the range of *D* follows from the Stokes–Einstein relation in free solution for a 4 nm fragment. The lower limit of *D* is an estimate for the restricted diffusion imposed by the nanostar meshwork, which is based on measurements of the diffusion of similarly sized objects within the bacterial cytoplasm^28^; this is likely a lower bound, since the bacterial cytoplasm is more dense in macromolecule (volume fraction *ϕ* ≈ 30%^29^) than the nanostar liquid (*ϕ* ≈ 1%^17^). Using these values, we find Pe ≈ 0.02–0.002, indicating diffusive transport dominates.

The small value of the Peclet number indicates that fragments generated within the DNA liquid execute diffusive random walks that terminate by stochastically exiting into either the vacuole or the surrounding bulk solution (see Fig. 4). We define *q* to be the rate of fragment capture by the vacuole relative to the total amount generated. We estimate *q* by solving the diffusion equation in steady state, assuming a homogeneous rate of fragment generation throughout the DNA liquid, and taking the vacuole and external interfaces to act as perfectly absorbing, concentric spheres (see Supplementary Discussion). In reality, the finite diffusivity of the fragments through the DNA liquid means that the vacuole is not perfectly absorbing (i.e., fragments can leak out of the vacuole); however, that the vacuoles grow at all indicates that fragments are largely retained on the relevant timescales, and the absorbing assumption is justified. Assuming *R*_v_ ≪ *R*_d_, the result of the diffusion modeling is *q* ≈ *R*_v_/(2*R*_d_) (see Supplementary Discussion). Given *M* enzymes dispersed throughout the DNA phase that each generate fragments at a rate *f*, the total rate of fragment generation is *Mf*, and the rate of fragment accumulation in the vacuole is

1 $$\dot{N}=\frac{1}{2}Mf\frac{{R}_{{{{{{\rm{v}}}}}}}}{{R}_{{{{{{\rm{d}}}}}}}}$$

where *N* is the number of fragments in the vacuole.

Fragment accumulation increases Π, the osmotic pressure in the vacuole, which we estimate using the van’t Hoff relation, Π ≈ *Nk*_B_*T*/*V*, given thermal energy *k*_B_*T* and vacuole volume *V*; this relation is appropriate for the dilute nature of the fragments in the vacuole as indicated by its relative darkness in the fluorescent images. Water is drawn into the vacuole until the swelling pressure, Π, is balanced by the Laplace pressure of the vacuole interface, leading to the constraint

2 $$\frac{N{k}_{{{{{{\rm{B}}}}}}}T}{V}=\frac{2\gamma }{{R}_{{{{{{\rm{v}}}}}}}}$$

where *γ* is the interfacial tension of the nanostar liquid. Assuming *R*_d_ is approximately constant in time (which is relevant for much of the vacuole growth curve; Fig. 2), and noting that $V=4\pi {R}_{{{{{{\rm{v}}}}}}}^{3}/3$, we can solve the coupled constraints, Eqs. (1) and (2), for the trajectories of *R*_v_ and *N* versus time, *t* :

3 $$	{R}_{{{{{{\rm{v}}}}}}}(t)=\frac{3{k}_{{{{{{\rm{B}}}}}}}TMf}{32\pi \gamma {R}_{{{{{{\rm{d}}}}}}}}t\\ 	 N(t)=\frac{3{k}_{{{{{{\rm{B}}}}}}}T{M}^{2}{f}^{2}}{128\pi {R}_{{{{{{\rm{d}}}}}}}^{2}\gamma }{t}^{2}$$

Both quantitative and qualitative predictions of this model are supported by experiments. In particular, the model predicts a linear growth of vacuole radius with time, as observed (Fig. 2). Further, quantitative predictions of the growth rate, based on estimates of the parameters in Eq. (3), are consistent with the measured growth rates. Specifically: The maximal catalytic rate of the Sma I enzyme was measured to be *f* = 0.4/s^30^. Surface tension was previously measured in roughly analogous salt concentrations, giving *γ* ≈ 4 μN/m^17^. A typical initial droplet radius is *R*_d_ ≈ 20 μm (Fig. 2). Our prior results indicate that, at the late times at which vacuoles appear, the concentration of enzymes in the liquid phase becomes similar to that in bulk^20^, corresponding to *c*_e_ ≈ 3 nM. Using this, we estimate there are typically $M=4\pi {R}_{{{{{{\rm{d}}}}}}}^{3}{c}_{{{{{{\rm{e}}}}}}}/3\, \approx \,6\times 1{0}^{4}$ enzymes in a 20 μm droplet. These parameter estimates are somewhat rough, due to differences in, e.g., the present solution conditions versus those in the prior works from which the estimates are taken. That said, using these numbers in Eq. (3) predicts a ≈36 nm/s vacuole growth rate, which is remarkably consistent with the range of measured slopes of 2–32 nm/s. These considerations demonstrate that the model accurately captures the microscopic physical effects that control the system’s behavior.

### The pressure and energetics driving motion

The model prediction for *N*(*t*) (Eq. (3)) indicates the fragment concentration in the vacuole, at timescales typical for vacuole popping (≈10^3^ s), is roughly *N*/*V* ≈ 0.1 μM. This is much less than the overlap concentration for the fragments (≈10 mM), confirming that the vacuole is a dilute solution, and validating the use of the van’t Hoff relation for osmotic pressure, which indicates Π ≈ 0.2 Pa. This value is consistent with the range of pressures estimated above from measurement of the burst velocities. We note that, while fragment osmotic pressure should be related to the mechanical pressure driving droplet motion, these are two distinct quantities. Prior work on the self-propulsion of microscale entities^31,32^ emphasizes that osmotic pressure, which is a free energy gradient that drives solvent flow into a mixture, is not capable of directly creating mechanical forces on an immersed object^32^. Instead, consistent with our analysis above, the propulsion is a result of the hydraulic pressure that results from the vacuole interfacial tension, which is loaded, like a spring, by the osmotic pressure.

From an energetic point of view, droplet motility is driven by a multi-stage, dissipative process. In the first stage, the free energy stored in the DNA backbone as phosphodiester bonds is released by the restriction enzymes, and converted to a relatively high fragment concentration in the vacuole. In the second stage, the fragment concentration is released by the popping event, and converted to the mechanical work driving motion. Using the experimental observations, and model estimates, the magnitude of each stage in this energy cascade can be estimated: The model predicts that, upon popping (*t* ≈ 10^3^ s), roughly *Mft* ≈ 2.4 × 10^7^ N double-stranded DNA cleavage events have occurred (with ≈20% of the resulting fragments accumulating in the vacuole). Hydrolysis (cleavage) of a single DNA strand yields Δ*G*_1_ = 5.3 kcal/mol of free energy^33^, so we estimate that double-strand cleavage yields roughly Δ*G*_2_ ≈ 10 kcal/mol, or ≈ 7 × 10^−20^ J per event. Thus the maximal free energy available from bond cleavage is *G*_bonds_ = *Mft*Δ*G*_2_ ≈ 1.7 × 10^−12^ J. The swollen vacuole stores energy predominantly in the excess fragment concentration, which can be estimated from the osmotic pressure and vacuole volume as *G*_vac_ ≈ Π*V* ≈ 7 × 10^−15^ J, given Π = 0.2 Pa and using a 20 μm radius vacuole (i.e., the typical size upon popping; Fig. 2). The mechanical work of droplet motion, *W* = *f*_burst_*d*, given *f*_burst_ ≈ 0.1 pN and typical distances *d* ≈ 35 μm (Fig. 3), is *W* ≈ 4 × 10^−18^ J.

The energetic analysis affirms that our picture of the process is thermodynamically consistent in that the estimated free energy decreases in each stage of the process, i.e., *G*_bonds_ > *G*_vac_ > *W*. Further, it indicates a high degree of inefficiency in each conversion step, with *G*_vac_/*G*_bonds_ ≈ 0.004, and *W*/*G*_vac_ ≈ 6 × 10^−4^. These inefficiencies are likely due to losses in energy transduction, including the loss of fragments to the bulk in the first conversion, and, in the second conversion, jetting power that is wasted by directing the droplet towards the glass surface rather than laterally.

## Discussion

We have used a model system to investigate active, dissipative dynamics within liquid-phase biomolecular droplets. Generally, the insights generated from our results show the power of combining sequence-engineered biomolecular phase separation and enzymatic coupling with precision measurements and modeling. More particularly, this work shows the existence of an enzyme-driven “bubbling” behavior that is associated with popping-driven droplet motility. A key qualitative result is that vacuole radii grows linearly with time for the entire observed duration until popping, encompassing a total radius increase of up to fivefold. We developed a model that captures this linear growth based on the diffusive capture by the vacuole of fragments generated by enzymes located in the DNA liquid phase (Eq. (1)), in combination with the mechanical balance of fragment osmotic pressure and the vacuole’s Laplace pressure (Eq. (2)). Remarkably, this model, using only independently estimated microscopic constants, predicts vacuole growth rates that are quantitatively consistent with those measured in experiment. Further, the model predicts vacuole osmotic pressures consistent with those estimated from the forces observed to drive droplet motion. Finally, the model output allows estimate of the scale of the dissipative energy cascade that powers motility.

It is of interest to compare the droplet motile behavior, as enabled by vacuole popping, to other motile microsystems. The typical droplet speeds, ≈50 nm/s, are similar to the speeds of ≈10 nm/s found recently for both living cells^34^ and artificial liposomes^35^ that are driven by osmotic pressure gradients across their lipid membranes. However, other artificial “microswimmers” have been reported that reach speeds of ≈10^4^ nm/s; these include a variety of motile droplet systems^36^, as well as solid particles that are driven by attached enzymes^37–39^. The relative lethargy of the present system is at least partially due to the slow enzymatic process it relies upon: the restriction enzyme used here has a turnover rate of order 1/s^30^, far less than the rates of 10^4^–10^6^/s of enzymes such as urease and catalase that are utilized in other enzymatically driven microswimmer systems^39,40^. Yet, our analysis indicates some improvement is possible by engineering the system to more efficiently convert energy: For example, it is of interest to create asymmetric systems, e.g., Janus-like droplet dimers^12,41^, where the enzymatic activity is localized to one side of the entity, permitting pore placements that lead to lateral, rather than vertical, jetting forces. While the potential utility of the popping-based motility reported here is as yet unclear, it has the virtue of being relatively well understood through our model; thus it could be both interesting and achievable to explore methods of improving this motility mechanism.

The physical picture presented here gives insight into the complex dynamics that can occur in biomolecular liquids driven by embedded active, enzymatic reactions, with implications for biological condensates, or synthetic droplet systems, that contain such non-equilibrium processes. The reaction used here is degradative (cleaving of DNA by a restriction enzyme); however, the interpretation supported by the model is that the vacuole behavior is driven by the enzymatic generation of diffusing fragments that accumulate in, and swell, the vacuole. Thus similar dynamics might result from other reactions embedded in condensates that generate solutes, notably including transcription, if the solute generation rate and condensate materials properties fall in the appropriate regime. This work indicates that the key materials properties are the mesh size and the interfacial tension of the biomolecular liquid. Here, the mesh size of the nanostar liquid (≈8 nm^27^) accomplishes two functions: it is small enough to largely contain the fragments in the vacuole, while still allowing diffusive transport of fragments that are generated in the liquid DNA phase. Notably, other macromolecular and biomolecular liquids have been shown to have similar mesh sizes^23,42–44^, indicating similar solute transport behaviors are possible in those condensates. The second key materials property is the low interfacial tension, ≈4 μN/m^17^, which lowers the Laplace pressure, and allows modest osmotic pressures (Π < 1 Pa) to create dramatic vacuole swelling effects. Other biomolecular liquids are known to have such low interfacial tensions^45,46^. Overall, this work demonstrates a range of dramatic condensate physical behaviors that can be related to specific materials properties that control the condensate’s response to embedded biochemical reactions; that the materials properties are widespread argues that the behaviors could also be widespread, with broad implications for the microscopic physical understanding of biomolecular condensates.

## Methods

### Experimental setup

Nanostar droplets of typically a few tens of μm in diameter, and labeled with the fluorophore Cy3, were prepared from DNA oligomers designed in NUPACK^26^ (sequences given in Supplementary Table 1) through a successive procedure of mixing (10 μM oligomer, including 0.1 μM of fluorophore-labeled oligomer, in a solution of 20 mM Tris, 50 mM K-acetate, and 10 mM Mg-acetate, pH 7.9), annealing (95 °C for 2 min, followed by cooling to 20 °C over 2 h), and incubation (rotated for 1 h at 20 °C), as in ref. ^20^. Once formed, droplets were added to a flow cell for fluorescent visualization. The restriction enzyme Sma I (New England Biolabs) was added to a final concentration 3–7 nM. Measurements of vacuole growth were carried out using droplets fixed lightly to the flow-cell surface; this was achieved using a hydrophobic glass surface (Sigmacote, Sigma) to which droplets were allowed to strongly adhere for 45 min, then weakening adhesion by adding BSA (0.1 mg/mL), see also ref. ^20^. Weakly adhered droplets had enzyme added after droplet fixation. Measurements of droplet motility were carried out by pre-treating the hydrophobic flow cell with BSA (0.1 mg/mL) so as to disallow adhesion, with enzyme added to the droplet solution prior to introduction to the flow cell. Experiments were carried out at 26 °C, and images were acquired using a Nikon Eclipse Ti Microscope outfitted with an Okolab Cage Incubator, a Lumencor Sola solid-state white-light excitation source, and a Chroma DSRed ET filter cube, with a ×4 objective and a pco.edge sCMOS camera.

### Image analysis

Vacuole and droplet radii were found by fitting the double-sphere function (Supplementary Discussion) to images. To estimate motility parameters, images of droplets were thresholded to identify the outer boundary of the droplet, and the intensity-weighted droplet centroid was calculated from the region inside the boundary so as to estimate the droplet center, and thus motion, from frame to frame. All calculations were carried out in Mathematica.

### Reporting summary

Further information on research design is available in the Nature Portfolio Reporting Summary linked to this article.

## Supplementary information

Supplementary information

Description of additional supplementary files

Supplementary Movie 1

Supplementary Movie 2

Supplementary Movie 3

Supplementary Movie 4

Supplementary Movie 5

Supplementary Movie 6

Reporting Summary

## Data Availability

Movies from Figs. 1 and 3 are provided in the Supplementary Information. Other data sets generated during the current study are available from the corresponding author on reasonable request. Source data are provided with this paper.

## Source data

Source data

## Code availability

The code used to analyze droplet and vacuole size (Fig. 2) has been deposited in the Dryad database under accession code 10.25349/D9X032. Other code generated during the current study are available from the corresponding author on reasonable request.
