# Supplementary material for: Vacuole dynamics and popping-based motility in liquid droplets of DNA
Source: Nat Commun. 2023 Jun 16;14:3574. doi: 10.1038/s41467-023-39175-0 (PMC10275875; doi:10.1038/s41467-023-39175-0)
Supplement: Supplementary file 1 — Supplementary information [file 41467_2023_39175_MOESM1_ESM.pdf]

# Supplementary Information for: Vacuole Dynamics and Popping-based Motility in Liquid Droplets of DNA

Omar A. Saleh, Sam Wilken, Todd M. Squires, Tim Liedl

May 12, 2023

This PDF file includes:
Supplementary Discussion.
Supplementary Table 1.
Supplementary Figure 1.

## Supplementary Discussion

### Double-sphere fitting function

To analyze vacuole growth dynamics (Fig. 2), droplet images were fit by a function describing the projection into two dimensions of a (solid) sphere (representing the fluorescent DNA within the droplet) with a second sphere subtracted from it (representing the relative lack of fluoresence of the vacuole). The resulting 'double-sphere' formula to describe the intensity, $I$, as a function of position, $(x, y)$, is as follows:

$$
\begin{aligned}
I(x,y) &= a + 2b\left[\sqrt{R_d^2 - (x-x_d)^2 - (y-y_d)^2} - \sqrt{R_v^2 - (x-x_v)^2 - (y-y_v)^2}\right], \\
&\qquad\qquad\qquad\qquad (x,y) \in \boldsymbol{D} \text{ and } (x,y) \in \boldsymbol{V} \\
&= a + 2b\left[\sqrt{R_d^2 - (x-x_d)^2 - (y-y_d)^2}\right], \qquad (x,y) \in \boldsymbol{D} \text{ and } (x,y) \notin \boldsymbol{V} \\
&= a, \qquad (x,y) \notin \boldsymbol{D} \text{ and } (x,y) \notin \boldsymbol{V} \qquad \text{(S1)}
\end{aligned}
$$

The parameters are the droplet and vacuole radii, $R_d$ and $R_v$; the droplet and vacuole center position, $(x_d, y_d)$ and $(x_v, y_v)$; and the variables describing the fluorescent intensity of the droplet, $b$, and the background, $a$. The conditions for using each equation describe whether or not the pixel at $(x, y)$ falls within the area $\boldsymbol{D}$ associated with the droplet, or the area $\boldsymbol{V}$ associated with the vacuole. That is, the conditions are related to the the following relations:

$$
\begin{aligned}
(x,y) \in \boldsymbol{D} &\implies R_d^2 - (x-x_d)^2 - (y-y_d)^2 > 0 \\
(x,y) \in \boldsymbol{V} &\implies R_v^2 - (x-x_v)^2 - (y-y_v)^2 > 0 \qquad \text{(S2)}
\end{aligned}
$$

## Modeling diffusive capture of fragments

Here, we set up and solve the diffusion equation that models the flux into the vacuole of fragments that are generated by enzymes within the DNA liquid phase, i.e. the model underlying Eq. 1 of the main text. We assume DNA fragments are generated homogeneously throughout the DNA liquid phase, consisting of a spherical droplet with an outer radius $R_d$ that contains a concentric internal vacuole of radius $R_v$. The fragments are created at a rate $g_0$ (with units fragments per volume per time), and diffuse within the droplet with diffusivity $D$. Fragments that reach the droplet radius $R_d$ are assumed lost to the bulk supernatant. Fragments that reach the vacuole radius $R_v$ are assumed to be absorbed into the vacuole. The DNA fragments thus obey the diffusion equation with a source,

$$\frac{\partial c}{\partial t} = D\nabla^2 c + g_0, \tag{S3}$$

with absorbing boundary conditions

$$c(R_d) = 0, \tag{S4}$$

$$c(R_v) = 0, \tag{S5}$$

where $c(r,t)$ is the fragment concentration at radius $r$ and time $t$. Assuming concentrations evolve slowly enough for the diffusive profiles to be quasi-steady gives

$$0 = D\nabla^2 c + g_0, \tag{S6}$$

with solution

$$c(r) = -\frac{g_0}{6D}\frac{(r-R_v)(r-R_d)(r+R_v+R_d)}{r}. \tag{S7}$$

The diffusive flux to the vacuole is given by

$$J_{\text{vac}} = -4\pi R_v^2 D \frac{dc}{dr}\bigg|_{r=R_V} = \frac{4\pi R_v^3}{3} g_0 \left(1 - \frac{R_d}{R_v}\frac{(R_v+R_d)}{2R_v}\right), \tag{S8}$$

the flux out of the drop is given by

$$J_{\text{drop}} = \frac{4\pi R_d^3}{3} g_0 \left(1 - \frac{R_v}{R_d}\frac{(R_v+R_d)}{2R_d}\right), \tag{S9}$$

from which it follows that the total flux out of the liquid phase matches the generation rate of fragments within the liquid volume,

$$J_{\text{drop}} + J_{\text{vac}} = \frac{4\pi g_0}{3}(R_d^3 - R_v^3). \tag{S10}$$

Of the fragments generated within the DNA liquid phase, the fraction that is absorbed by the vacuole, $q$, is given by

$$q = \frac{J_{\text{vac}}}{J_{\text{total}}} = \frac{\lambda(1+2\lambda)}{2(1+\lambda+\lambda^2)} \tag{S11}$$

where

$$\lambda = \frac{R_v}{R_d} \tag{S12}$$

is the radius of the vacuole relative to that of the drop. For small vacuoles, the fraction of fragments absorbed by the vacuole,

$$q \approx \frac{\lambda}{2} + \frac{\lambda^2}{2} + \mathcal{O}(\lambda^3) \qquad \text{(S13)}$$

is approximately one half of the ratio of the radii, which gives Eq. 1 of the main text.

| *Nanostar* | *Oligomer* | *Sequence* |
|---|---|---|
| Weak tetramer | OS1 | *CGATCG* t GCGGTGACCCGGGTTGACCG tt GCGCCCTGGAAAGGCCACCC |
| | OS4 | *CGATCG* t GGGTGGCCTTTCCAGGGCGC tt AGGGATTCCCGGGAATTCTC |
| | OS5 | *CGATCG* t GAGAATTCCCGGGAATCCCT tt GGTAGGACCCGGGCTGACCC |
| | OS6 | *CGATCG* t GGGTCAGCCCGGGTCCTACC tt CGGTCAACCCGGGTCACCGC |
| | | |
| Trimer | OSTri-1B | CGCCAGTCCCGGGAGCCTCG tt CGCGCTGGGTGCCGCATGCG t *TGGCCA* |
| | OSTri-2B | CGCATGCGGCACCCAGCGCG tt CGGTGGTCCCGGGACGTCCG t *TGGCCA* |
| | OSTri-3B | CGGACGTCCCGGGACCACCG tt CGAGGCTCCCGGGACTGGCG t *TGGCCA* |
| | | |
| Strong tetramer | OS1-B | *ACCGGT* t GCGGTGACCCGGGTTGACCG tt GCGCCCTGGAAAGGCCACCC |
| | OS4-B | *ACCGGT* t GGGTGGCCTTTCCAGGGCGC tt AGGGATTCCCGGGAATTCTC |
| | OS5-B | *ACCGGT* t GAGAATTCCCGGGAATCCCT tt GGTAGGACCCGGGCTGACCC |
| | OS6-B | *ACCGGT* t GGGTCAGCCCGGGTCCTACC tt CGGTCAACCCGGGTCACCGC |
| | | |

**Supplementary Table 1.** DNA sequences used in this work. As in our prior work, three different types of DNA nanostars were used, with names as given in the table. As noted in the main text, no significant difference was observed in vacuole dynamics between different types of nanostars. Each nanostar consists of 3 or 4 double-stranded DNA arms joined at a junction containing unpaired bases; at the end distal to the junction, each arm is terminated with an unpaired base and a sticky-end sequence that mediates nanostar condensation. In the table, sticky-end sequences are italicized and bases left unpaired within the nanostar are given in lowercase, while the remaining (capital, non-italicized) bases form the double-stranded DNA arms. Within the arms, Sma I recognition sequences are underlined. The spaces within each sequence only serve to clarify transitions between different portions of the nanostar, and have no structural or chemical meaning.

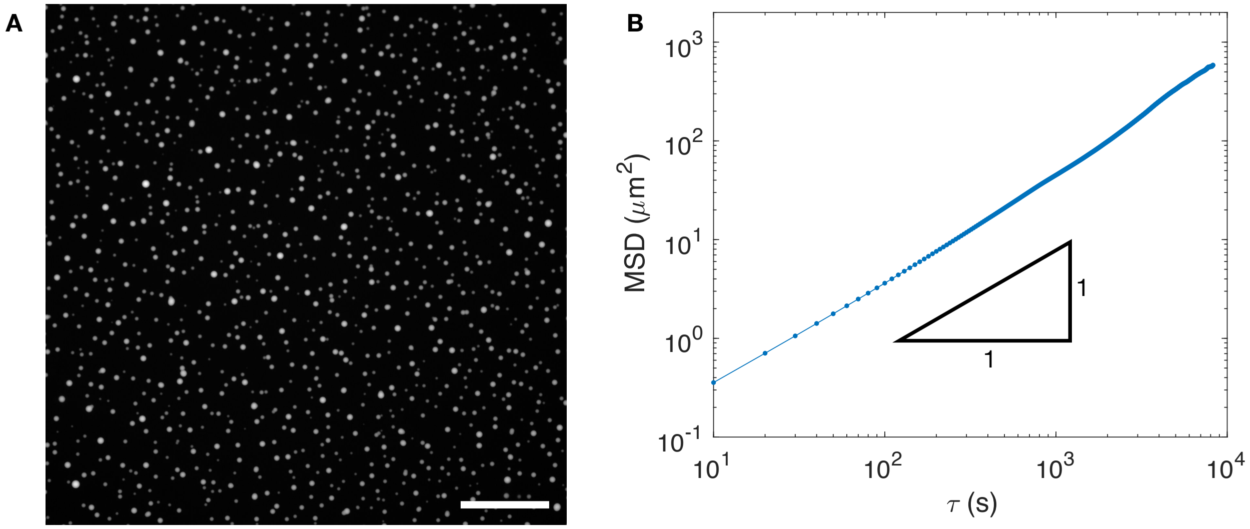


**Supplementary Figure 1.** Analysis of thermal (Brownian) diffusion of nanostar droplets, in the absence of enzymes. **A** One frame from a movie of nanostar droplets sedimented on a glass surface, as imaged on a fluorescent microscope. Drops are imaged for 800 frames, with 10 seconds between frames. Scale bar: 200 $\mu$m. **B** From the movie, we quantify the trajectories of droplets with with radius near $a = 8$ $\mu$m, and calculate the mean squared displacement, $\text{MSD} = \langle [x(t) - x(t-\tau)]^2 + [y(t) - y(t-\tau)]^2 \rangle$. The MSD versus lag time, $\tau$, is shown for 31 such droplets. The linearity of the plot indicates diffusive behavior. Given the two-dimensional nature of the diffusion, we expect $\text{MSD}_{2D} = 4D\tau$, and linear fitting gives a diffusion constant $D = 0.01$ $\mu\text{m}^2/\text{s}$. The Stokes-Einstein relation for a sphere in a viscous liquid in free space estimates the diffusion constant as $D = \frac{k_B T}{6\pi\eta a} = 0.027$ $\mu\text{m}^2/\text{s}$, given $a = 8$ $\mu$m. Thus our analysis indicates there is a roughly 3-fold increase of drag for thermal motion, relative to the free-space expectation, which we attribute to the hydrodynamic effects of the nearby glass surface.
