# Supplementary material for: Vacuole dynamics and popping-based motility in liquid droplets of DNA
Source: Nat Commun. 2023 Jun 16;14:3574. doi: 10.1038/s41467-023-39175-0 (PMC10275875; doi:10.1038/s41467-023-39175-0)
Supplement: Supplementary file 2 — Description of additional supplementary files [file 41467_2023_39175_MOESM2_ESM.pdf]

Description of additional supplementary files

Supplementary Movie 1: Time series movie showing two consecutive vacuole growth events in a single droplet. The movie is an animation of the data shown in panel B of main-text Fig 1. Time in minutes is shown on each frame, along with a 40 micron scale bar.

Supplementary Movie 2: Time series movie demonstrating popping-driven motility of a DNA droplet, corresponding to column A of main-text Fig. 3. Time in minutes is shown on each frame, along with an 80 micron scale bar.

Supplementary Movie 3: Time series movie demonstrating popping-driven motility of a DNA droplet, corresponding to column B of main-text Fig. 3. Time in minutes is shown on each frame, along with an 80 micron scale bar.

Supplementary Movie 4: Time series movie demonstrating popping-driven motility of a DNA droplet, corresponding to column C of main-text Fig. 3. Time in minutes is shown on each frame, along with an 80 micron scale bar.

Supplementary Movie 5: Time series movie demonstrating popping-driven motility of a DNA droplet, corresponding to column D of main-text Fig. 3. Time in minutes is shown on each frame, along with an 80 micron scale bar.

Supplementary Movie 6: Time series movie demonstrating popping-driven motility of a DNA droplet, corresponding to column E of main-text Fig. 3. Time in minutes is shown on each frame, along with an 80 micron scale bar.
