# Supplementary figures and images for: Vacuole dynamics and popping-based motility in liquid droplets of DNA
Source: Nat Commun. 2023 Jun 16;14:3574. doi: 10.1038/s41467-023-39175-0 (PMC10275875; doi:10.1038/s41467-023-39175-0)

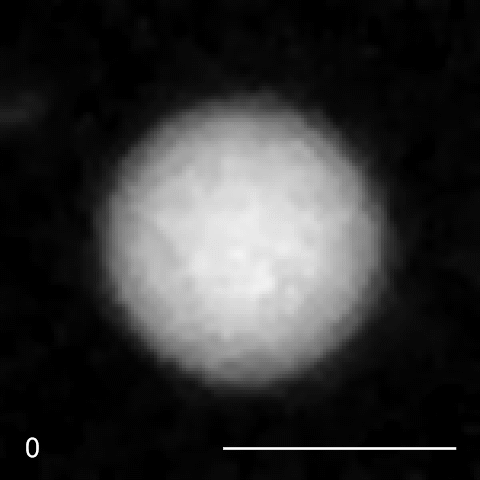

Supplement: Supplementary file 3 — Supplementary Movie 1 [file 41467_2023_39175_MOESM3_ESM.gif]

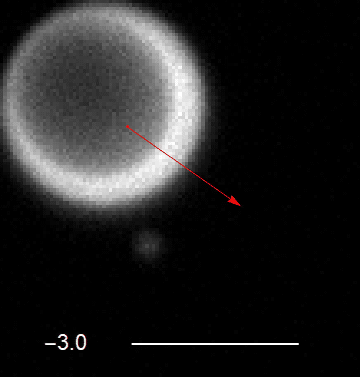

Supplement: Supplementary file 4 — Supplementary Movie 2 [file 41467_2023_39175_MOESM4_ESM.gif]

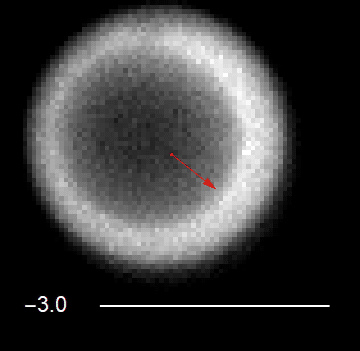

Supplement: Supplementary file 5 — Supplementary Movie 3 [file 41467_2023_39175_MOESM5_ESM.gif]

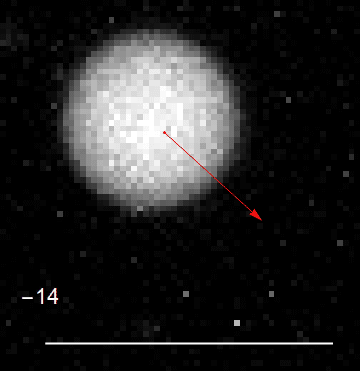

Supplement: Supplementary file 6 — Supplementary Movie 4 [file 41467_2023_39175_MOESM6_ESM.gif]

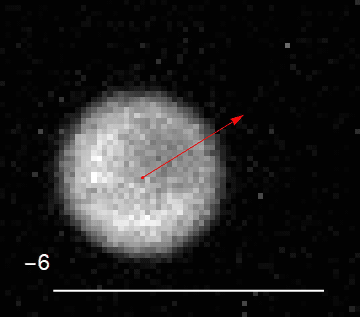

Supplement: Supplementary file 7 — Supplementary Movie 5 [file 41467_2023_39175_MOESM7_ESM.gif]

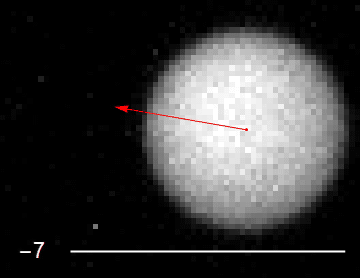

Supplement: Supplementary file 8 — Supplementary Movie 6 [file 41467_2023_39175_MOESM8_ESM.gif]
